# Outcome of isolated fetal choroid plexus cyst detected in prenatal sonography among infertile patients referred to Royan Institute: A 3-year study

**Published:** 2015-09

**Authors:** Shohreh Irani, Firoozeh Ahmadi, Maryam Javam, Ahmad Vosough Taghi dizaj, Fatemeh Niknejad

**Affiliations:** 1 *Department of Reproductive Imaging, Reproductive Biomedicine Research Center, Royan Institute for Reproductive Biomedicine, ACECR, Tehran, Iran.*; 2 *Department of Epidemiology and Reproductive Health, Reproductive Epidemiology Research Center, Royan Institute for Reproductive Biomedicine, ACECR, Tehran, Iran.*

**Keywords:** *Fetus*, *Anomaly*, *Ultrasonography*

## Abstract

**Background:**

Several studies have assessed the correlation of fetal choroid plexus cyst (CPC) and the risk of congenital anomalies, but few ones have discussed isolated CPC (with no other abnormal sonographic finding).

**Objective:**

The aim of this study was to determine the outcome of isolated fetal choroid plexus cyst and to specify its clinical significance.

**Materials and Methods:**

This cross sectional study was carried out at Royan Institute in Tehran, Iran, between April 2009 and December 2012. All prenatal sonographies in this period of time were assessed using a computerized database and fetuses who had isolated CPC were recruited in the study. Sonography reports, mother serum screening test results, fetal echocardiography and amniocentesis were evaluated until birth. A follow-up phone call was made to all individuals to learn about the neonatal outcomes.

**Results:**

Overall, 6240 prenatal sonographies were performed in this setting during this period. Isolated CPC was detected in 64 fetuses. The results of double test (N=30), triple test (N=5) and fetal echocardiography (N =24) were normal. Quadruple test result showed 3 abnormal out of 29 cases that all had normal karyotypes. Four samples were dropped out due to premature rupture of membranes (N=3) and intrauterine fetal death (N=1). It was found that the outcomes of all remaining fetuses (N=60) were normal and no anomaly ones were seen until birth.

**Conclusion:**

Isolated CPC is a benign regressive condition with no clinical significance.

## Introduction

Choroid plexus cysts (CPCs), small fluid-filled structures constituted from the lateral ventricles of the fetus brain, are found in second trimester screening sonography (1-5). These cysts may be single/multiple or unilateral/bilateral (4, 6). They are detected in 1-3.6% of all fetuses, while 90% are resolved by 26-28th week of gestation (3, 4, 6-11). Most of choroid plexus cysts are considered “a benign condition” resulted from “trapped CSF within rapid growing ventricles”, but they may be caused by presence of a chromosomal abnormality, mostly Trisomy18 (T18) (4, 9).

The correlation between CPC and Trisomy18 was first proposed in 1984 (3, 8, 9, 12-14), and CPC was observed in %30-50 of Trisomy18 cases(8, 14). The likelihood of aneuploidy will rise if other structural anomalies besides CPC are detected in ultrasound examination (such as heart, hand defects, etc.), or if mother serum screening indicates higher risk of abnormality (6, 9, 12). Several studies have assessed the correlation of T18 and CPC in these situations, but few have discussed about isolated CPC (with no other abnormal sonographic findings) (1-3, 5, 12, 15-18). Thus, significance of isolated CPC is unclear, and releasing information to mothers whose fetus had an isolated CPC detected through ultrasound examination has been also debated (3, 8, 9, 12). Thus, this study was designed to evaluate the outcome of isolated CPC detected in screening sonography and to specify its clinical significance.

## Materials and methods

This cross-sectional descriptive study was conducted on 6240 pregnant women undergoing prenatal investigation in our imaging department at Royan Institute, Tehran, Iran, between April 2010 and September 2012. All sonography reports had been documented by a computerized database. Medical records of laboratory and genetic tests had been also documented in this database.

To collect data, it was revised ultrasound reports of all 6240 pregnancies to find out fetuses with CPC. We carefully noted whether there was any document of major anomaly in the cerebellum, lateral cerebral ventricles, spine, facial profile, four-chamber view of the heart, extremities, abdomen, umbilical cord, kidneys, bladder and/or any sonographic soft markers. If there was any abnormality, the patient was excluded from the study. Then, results of double test, triple test, quadruple test, fetal echocardiography and amniocentesis (which were performed as ordered by a perinatologist) were reviewed. To investigate the karyotype of fetuses, a 20-gauge needle was inserted into the uterus with “real time ultrasound guidance”, which allowed continuous control of the needle. Most commonly, 15ml of amniotic fluid was aspirated for harvesting and analysis of the DNA in the laboratory. After 2 weeks of cell division, appropriate number of cells were studied microscopically, on the basis of Giemsa-Trypsi -Giemsa (GTG) technique at 400-500 band resolution, which revealed the number of chromosomes.

Demographic history (to detect possible risk factors), ultrasound examinations and results of para-clinical tests were written in designed questionnaires. Since all newborns were clinically examined by a pediatrician right after birth, it was made follow-up calls to all individuals to learn about the neonatal outcomes.

This study was approved by a research ethics committee and institutional review board at Royan Institute, Tehran, Iran, and informed written consent was obtained from the patients before using their records.

**Patients**

This study assessed the infertile patients who became pregnant following assisted reproductive technologies (ARTs), induction/intra uterine insemination (IUI) method or spontaneously (between periods of treatment). The total numbers of 6240 prenatal examinations were performed by two board-certified radiologists using Aloka-α10 equipment (convex probe, 3.5-5Mhtz-Japan) during a period of 30 months. The inclusion criteria were as follows: detection of isolated CPC by sonography, no genetic disorder in parents, and mother aged less than 40 years. Sixty four patients meeting the criteria were recruited in the study.

**Statistical analysis**

In this study, all information obtained by questionnaires was entered into statistical package for the social sciences, version 16.0, SPSS Inc, Chicago, Illinois, USA (SPSS). Descriptive statistics were used to calculate the frequency, central tendency (mean, median & mode) and dispersion (range, variance, SD, maximum & minimum) for each variable.

## Results

In a period between April 2010 and September 2012, prenatal screening sonography was performed on 6240 pregnant women in the imaging department. Totally, 64 fetuses with isolated CPC were found during this period (47 single fetuses, 15 twins and 2 triplets).

The mean age of mothers was 30.25±4.05 years. The average infertility duration was 5.44 ± 0.44 years (four women experienced secondary infertility). Regarding the reason of infertility, the male factor was detected in 25 cases (39.6%), whereas female factors were found in 28 cases (43.75%). In other 18 cases (17.18%), there were multifactorial reasons. Pregnancy in 31 cases (%48.3) occurred using in vitro fertilization (IVF) or intra cytoplasm sperm injection (ICSI), in 17 women (26.6%) occurred using IUI, in 3 cases (4.7%) occurred following “frozen thawed embryo transfer” method and in 13 women (20.3%) occurred spontaneously between the periods of treatment.

There were 64 fetuses with CPCs, and the ratio of males to females was 1:1. CPCs were visualized bilaterally in 40 cases (62.5%) and unilaterally in other 24 cases (37.5%). Additionally, among twine or triple pregnancies, CPC was found only in one of the fetuses. The size of the cysts ranged between 5mm to 15mm with an average of 5.71± 0.29 mm. CPC was first diagnosed at the 14th week of gestation, but most of the cysts were detected at 17th week of gestation (probably due to lack of earlier exam).

Results of other screening investigations were as follows (Table I).

**Table I:** Frequency of normal and abnormal laboratory test results in fetuses with isolated choroid plexus cyst

	**Total**	**Normal**	**Abnormal**
Double test	30(100%)	30(100%)	0
Triple test	5(100%)	5(100%)	0
Quadruple test	29(89.6%)	26(100%)	3(10.4%)
Fetal echochardiography	24(100%)	24(100%)	0
Amniocentesis	8(100%)	8(100%)	0

**Figure 1:**
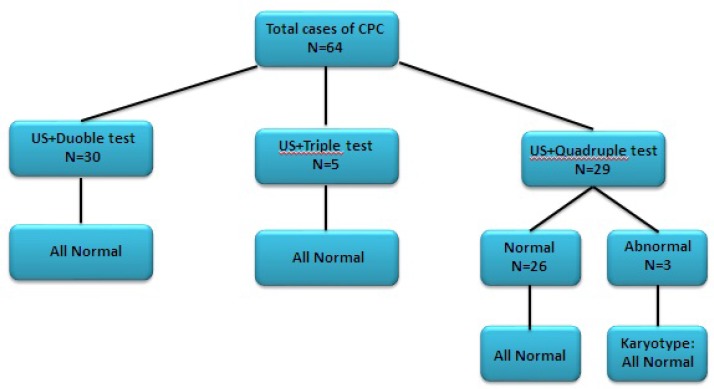
Mother Serum Screening correlated with ultrasound evaluation of isolated choroid plexus cyst cases

Thirty women had been assessed by double test and 5 women by triple test; all results were normal. In addition, quadruple test was done for 29 fetuses, which demonstrated normal result in 26 cases. Among 3 fetuses with abnormal quadruple test results, the risk factors of Down syndrome were 1/33 and 1/24 in 2 fetuses, and alpha-fetoprotein level was higher than normal range in third one. However, amniocentesis did not show any abnormality for them. Fetal echocardiography was performed for 24 cases (37.5%) that all were normal. Amniocentesis was done for 8 fetuses with abnormal quadruple test or positive family history, and all had normal karyotype (Figure 1).

**Figure 2:**
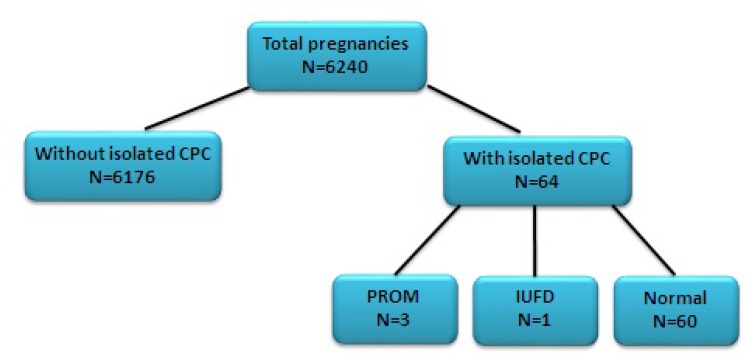
Outcome of isolated fetal choroid plexus cyst cases

During the research, four patients were excluded due to premature rupture of membranes (PROM) (N=3) and intrauterine fetal death (IUFD) (N=1). CPCs gradually disappeared by 25th week in all other 60 fetuses and no anomaly was seen in follow-up sonographic examinations. Follow-up phone calls revealed that all the newborns were normal, even in fetuses with previous CPC larger than 10mm or those with abnormal mother serum screening results (Figure 2).

## Discussion

In this study, the prevalence of isolated CPC among 6240 pregnant women during 30 months was 1.02%. In a study conducted by Cheng et al. on 7795 pregnancies, overall incidence of CPC was 1.25%, but isolated CPC was found in 1.05% of their samples (1). Similarly, the prevalence of isolated CPC in the study of Demasio et al. and kupferminc et al. were 0.95% and 1.07%, respectively , while it was 2.14% in the study conducted by Bronsteen et al. and 2.45% in the study of Coco et al. (17,18,12,2). 

The initial time of sonographic detection of CPC in our study was 14^th^ week of gestation according to last menstrual period (LMP), whereas it was first diagnosed in 15^th^ week by Bronsteen and colleagues (12). Mean age of diagnosis in this study was 17^th^ week in comparison with 19.2^th^ week in the study of Bronsteen et al. (12). All CPCs were resolved by 25^th^ week in our patients while Dipietro and colleagues reported that CPC regressed by 28^th^ week in their study (3).

Several studies have evaluated the correlation between CPC and T18 or other chromosomal abnormalities, but few studies have assessed the risk of isolated CPC. Thus, there is a controversy among researchers and practitioners about the risk factors of isolated CPC and about releasing information to parents (3, 8, 9, 12). Most researchers have indicated that isolated CPC is not associated with higher risk of aneuploidy, whereas few studies have demonstrated a correlation between isolated CPC and chromosomal abnormalities (16, 18). Table II summarizes some studies on CPC, isolated or with other US findings, and their findings about isolated CPC.

**Table II:** Comparison of some studies about choroid plexus cyst

	**Type of Study**	**Total Patients**	**Prevalence of CPC**	**isolated CPC**	**Gestational age(week)**	**Other investigations**	**Outcome**
This Study	Cross sectional	6240	-	64(1.02%)	14th-birth	Mother Serum Screening, Fetal Echocardiography	All NL
Dipietro et al (2006)	Case Control	102	-	35(-)	18-22	Neurobehavioral development of fetuses	All NL
Cheng et al (2004)	Cross sectional	7795	98(1.25%)	82(1.05%)	16-22	NT	All NL
Coco et al (2004)	Cross sectional	12672	366(2.88%)	311(2.45%)	16-23	-	All NL
Bronsteen et al (2004)	Cross sectional	49435	1209(2.3%)	1060(2.14%)	15-25	-	All NL
Demasio et al (2002)	Meta analysis	106732	1235(1.2%)	1017(0.95%)	NA	-	All NL
Sullivan et al (1999)	Cross sectional	128	NA	112(-)	18-22	Triple test / serum αFP	1 T18
Ostlere et al (1999)	Cross sectional	11700	100(0.085%)	NA	NA	-	3 T18 & 1 Syndaktyly (all with other US findings)
Kupferminc et al(1994)	prospective	9100	102(1.12%)	98(1.07%)	NA	NA	1 T18, 2 T21

NA: Not Available

NL: Normal

CPC: Choroid plexus cyst

Our study indicated that all isolated CPCs detected by ultrasound examination were resolved, and no abnormality was found in them. In addition, follow up phone calls showed neonatal health due to no report of anomaly by pediatrician right after birth. Similarly, in a study conducted by Coco et al. on 12672 pregnancies in a period of 5 years, 366 CPC cases were detected among whom 311 cases had isolated CPC (2). All isolated CPC cases were normal and no abnormality was found in them. Bronsteen and colleagues assessed 49435 fetuses aged 16-25 weeks and discovered 1209 CPC cases during 11 years. Of which, 1060 CPCs were isolated that all were normal, which is in agreement with the findings (12). Cheng et al. carried out a follow-up evaluation of CPC, nuchal translucency (NT) and other sonographic markers of aneuploidy (particularly T18) in 7795 pregnancies until birth. CPC was present in 98 fetuses as follows: 82 cases with isolated CPC and 16 cases with enlarged NT or other soft markers). All isolated CPCs had good prognosis and no abnormality was seen among them. Their study demonstrated that isolated CPC shows good outcomes, which confirms the current findings (1).

Lopez et al. believe that “when CPC is detected by sonography, amniocentesis or chorionic villus sampling (CVS) will be required if mother serum screening is abnormal” (9). In this study, there were only 3 abnormal quadruple test results, but all had normal karyotype. In a study conducted by Sullivan et al. on 128 fetuses with CPC, mother serum screening (triple test and/or αFP level) was compared to amniocentesis results and physical examination after birth. Their findings demonstrated that among 112 fetuses with isolated CPC, mother serum screening was positive in 22 cases (19.6%) among whom just two fetuses had T18. Although one fetus with T18 was detected among other 90 cases with normal serum screening result, this patient had been evaluated by means of single αFP screening (not triple test). They concluded that triple test adjunct to ultrasound screening is a reliable method to find out high risk fetuses with isolated CPC, whereas amniocentesis is need to be done only in cases with abnormal serum screening (16).

In contrast with these findings, Kupferminc et al. investigated 98 fetuses with isolated CPC among whom 75 fetuses were evaluated by karyotyping. They found 2 cases of T21 and one case of T18 and concluded that amniocentesis was required to be proposed to all patients with isolated CPC (18). In comparison, all fetuses in the present study were normal even those with positive serum screening. However, Kupferminc and colleagues did not assess mother serum screening in their study (18). Fetuses with abnormal karyotype often cause positive serum screening, which can be a helpful sign to detect high risk fetuses.

To the best of our knowledge, there has been no previous study that investigated CPC among the infertile population. Published reports on CPC among normal pregnant women suggested that isolated CPC were correlated with no chromosomal abnormality. This study about infertile population was in agreement with those papers.

To sum up, with respect to the mentioned findings, it can be concluded that isolated CPC is a benign regressive condition with no clinical significance, especially when mother serum screening is in normal range. Regarding releasing information to parents, they could be informed of absolute regression of the cyst by 25-28th week of gestation. They could get assured of their fetus heath, particularly when mother serum screening is negative. Further investigations such as amniocentesis are helpful in situations in which serum screening reveals higher risk of abnormalities.
